# The Clinical Characteristics, Carbapenem Resistance, and Outcome of *Acinetobacter* Bacteremia According to Genospecies

**DOI:** 10.1371/journal.pone.0065026

**Published:** 2013-06-03

**Authors:** Kyung-Hwa Park, Jong-Hee Shin, Seung Yeop Lee, Soo Hyun Kim, Mi Ok Jang, Seung-Ji Kang, Sook-In Jung, Eun-Kyung Chung, Kwan Soo Ko, Hee-Chang Jang

**Affiliations:** 1 Department of Infectious Diseases, Chonnam National University Medical School, Gwang-ju, Republic of Korea; 2 Department of Laboratory Medicine, Chonnam National University Medical School, Gwang-ju, Republic of Korea; 3 Department of Medical Education, Chonnam National University Medical School, Gwang-ju, Republic of Korea; 4 Department of Molecular Cell Biology, Sungkyunkwan University School of Medicine, Suwon, Republic of Korea; University of Padova, Medical School, Italy

## Abstract

**Background:**

Few clinical data are available on the relationship between genospecies and outcome of *Acinetobacter* bacteremia, and the results are inconsistent. We performed this study to evaluate the relationship between genospecies and the outcome of *Acinetobacter* bacteremia.

**Methods:**

Clinical data from 180 patients who had *Acinetobacter* bacteremia from 2003 to 2010 were reviewed retrospectively. The genospecies were identified by *rpoB* gene sequence analysis. The clinical features and outcomes of 90 patients with *A. baumannii* bacteremia were compared to those of 90 patients with non-*baumannii Acinetobacter* bacteremia (60 with *A. nosocomialis*, 17 with *Acinetobacter* species “close to 13 TU”, 11 with *A. pittii*, and two with *A. calcoaceticus*).

**Results:**

*A. baumannii* bacteremia was associated with intensive care unit-onset, mechanical ventilation, pneumonia, carbapenem resistance, and higher APACHE II scores, compared to non-*baumannii Acinetobacter* bacteremia (*P*<0.05). In univariate analyses, age, pneumonia, multidrug resistance, carbapenem resistance, inappropriate empirical antibiotics, higher APACHE II scores, and *A. baumannii* genospecies were risk factors for mortality (*P*<0.05). Multivariate analysis revealed *A. baumannii* genospecies (OR, 3.60; 95% CI, 1.56–8.33), age, pneumonia, and higher APACHE II scores to be independent risk factors for mortality (*P*<0.05).

**Conclusion:**

*A. baumannii* genospecies was an independent risk factor for mortality in patients with *Acinetobacter* bacteremia. Our results emphasize the importance of correct species identification of *Acinetobacter* blood isolates.

## Introduction

The spread of multidrug-resistant (MDR) *Acinetobacter* strains among critically ill, hospitalized patients, and subsequent epidemics, have become an increasing cause for concern [1], [2]. These organisms are one of the most common causes of infection in patients in the intensive care unit (ICU) and are associated with a greater risk of hospital death by causing catheter-related infection, pneumonia, urinary tract infection, wound infection, and primary bacteremia in patients with critical illness [3]–[6]. The organisms’ ability to accumulate diverse mechanisms of resistance limits therapeutic agents and makes the infection difficult to treat [7].

To date, more than 30 *Acinetobacter* species have been described [8]. Among these, *Acinetobacter baumannii* (genotype 2) is the most commonly found in clinical specimens. However, recently, infection caused by other *Acinetobacter* species belonging to the *A. calcoaceticus-A. baumannii* (ACB) complex, including *A. nosocomialis* (known also as 13 TU), *A. calcoaceticus* (genotype 1), *A. pittii* (genotype 3), and *Acinetobacter* genomic species “close to 13 TU”, has caused concern [9], [10]. Although these are increasingly reported as human pathogens with the introduction of genotyping, it is impossible to distinguish these from *A. baumannii* by automation systems or phenotypic tests [9], [11].

For these reasons, it is important to know whether or not the clinical features, risk factors, and outcomes differ according to species. To date, few studies have been published that evaluate this matter using genotypic assays in patients with *Acinetobacter* bacteremia [12]–[14]; however, among these reports, the influence of genospecies on outcome is inconsistent. Thus, we compared the clinical features, antimicrobial resistance, and outcome of bacteremia caused by *A. baumannii versus* non-*baumannii* ACB complex strains.

## Patients and Methods

### Ethics

This study was approved by the Institutional Review Board of Chonnam National University Hospital. A waiver of consent was granted given the retrospective nature of the project.

### Patients

All adult patients (age, ≥16 years) with bacteremia caused by ACB complex who were admitted to Chonnam National University Hospital (1000 beds; Gwang-ju, Republic of Korea) from January 2003 to December 2010 were included. Only patients with their first episode of *Acinetobacter* bacteremia were included.

### Data Collection

Demographic and clinical data were collected by reviewing the electronic medical records of the patients and included comorbid conditions, duration of ICU and hospital stay, use of a ventilator, and use of central venous catheters at the time of bacteremia onset. The severity of patient infection was evaluated using Acute Physiology and Chronic Health Evaluation (APACHE) II scores within 24 h of bacteremia onset.

### Microbiological Tests

*Acinetobacter* was identified using the automated system Vitek 2 (bioMérieux, Marcy l’Etoile, France). *Acinetobacter* species were determined on the basis of partial *rpoB* gene sequences (468 bp) detected using the primers Ac1055F (GTGATAARATGGCBGGTCGT) and Ac1598R (CGBGCRTGCATYTTGTCRT), as described by us previously [15], [16]. *In vitro* susceptibility testing was conducted using Vitek 2 (bioMérieux).

### Definitions

Multidrug resistance (MDR) was defined if an isolate showed resistance to more than one of the following five antimicrobial agents: imipenem, ciprofloxacin, amikacin, cefepime, and piperacillin/tazobactam, in accordance with Paterson’s suggestion [17]. Appropriate empirical antibiotics was defined as the use of at least one antibiotic to which the *Acinetobacter* strain was susceptible within 48 days from the onset of bacteremia. Mortality was defined as *Acinetobacter*-related in the absence of another definite cause of death [18].

### Statistical Analysis

Categorical variables were compared using Fisher’s exact test or the Pearson χ2 test, and continuous variables were compared using Student’s *t*-test or Mann-Whitney rank sum test, as appropriate. Variables with *P* values ≤0.10 in the univariate analysis were included in the multivariate analysis. Multivariate analyses were performed using a logistic regression model in the backward stepwise conditional manner. All tests of significance were two-tailed, and *P* values ≤0.05 were deemed to indicate statistical significance. Statistical analyses of the data were performed using SPSS software (version 19.0; SPSS Inc., Chicago, IL, USA).

## Results

### Study Population and Genospecies Distribution

During the study period, we identified 180 isolates of the ACB complex, including 90 (50%) *A. baumannii*, 60 (33%) *A. nosocomialis*, 17 (9%) *Acinetobacter* genomic species “close to 13 TU”, 11 (6%) *A. pittii*, and two (1%) *A. calcoaceticus.*

### Comparison of the Clinical Features of Bacteremia Caused by *A. baumannii versus* Non-baumannii ACB Complex

The clinical features of bacteremia caused by *A. baumannii* (n = 90) and non-*baumannii* ACB complex (n = 90) bacteria are shown in Table 1. Demographic data were not different between the two groups. ICU onset, mechanical ventilation, and underlying chronic liver disease were risk factors for *A. baumannii* bacteremia, compared to non-*baumannii Acinetobacter* bacteremia (*P*<0.05). Pneumonia, MDR, and carbapenem resistance were more commonly found in *A. baumannii* bacteremia, while primary bacteremia was more commonly caused by non-*baumannii Acinetobacter* (*P*<0.05). No significant difference in clinical features was observed between bacteremia caused by *A. nosocomialis*, *Acinetobacter* species “close to 13 TU”, and *A. pittii* (data not shown).

**Table 1:** Comparison of the clinical features of bacteremia caused by *A. baumannii* (n = 90) or non-*baumannii* ACB complex (n = 90).

Variables	No. (%) of patients with bacteremia caused by		*P* value
	*Acinetobacter baumannii* (n = 90)	Non- *baumannii* ACB complex (n = 90)	
Demographic data			
Age^a^	60±17	58±18	0.46
Male gender	62 (69)	53 (59)	0.16
Underlying illness			
Ischemic heart disease	25 (28)	16 (18)	0.11
Trauma	13 (14)	16 (18)	0.54
Diabetes mellitus	11 (12)	14 (16)	0.52
Cancer	8 (9)	12 (13)	0.34
Cerebrovascular accident	7 (8)	8 (9)	0.79
Chronic liver disease	14 (16)	3 (3)	0.01
End-stage renal disease	4 (4)	4 (4)	>0.99
Benign biliary disease	7 (8)	7 (8)	>0.99
Primary site of infection			
Vascular catheter	27 (30)	27 (30)	>0.99
Pneumonia	29 (32)	16 (18)	0.03
Primary bacteremia	8 (9)	18 (20)	0.03
Intra-abdominal	12 (13)	9 (10)	0.49
Urinary tract	3 (3)	7 (8)	0.33
Soft tissue or wound	7 (8)	7 (8)	>0.99
Other characteristics			
Length of hospitalization (day)^b,^ c	12 (7, 21)	11 (7, 22)	0.90
ICU stay at culture	77 (86)	57 (63)	0.001
Mechanical ventilation	64 (71)	44 (49)	0.002
MDR	69 (77)	29 (32)	<0.001
Carbapenem-resistance	51 (57)	12 (16)	<0.001
APACHE II score^a^	18±7	17±8	0.30
Outcomes			
30-day mortality	35 (39)	14 (16)	<0.001
Acinetobacter-related mortality	33 (37)	11 (12)	<0.001

ACB, *A. calcoaceticus-A. baumannii*; ICU, intensive care unit; MDR, multidrug resistance; APACHE, acute physiology and chronic health evaluation.

Continuous variables were expressed as means ± SDs ^a^ or medians (IQRs)^ b^ and were compared by the Student’s *t* test^ a^ or Mann-Whitney U test^ b^.

c Until the onset of *Acinetobacter* bacteremia.

### Risk Factors for 30-day Mortality in Patients with *Acinetobacter* Bacteremia

Risk factors for 30-day mortality in the 180 patients with *Acinetobacter* bacteremia are shown in Table 2. In the univariate analysis, *A. baumannii* genospecies, age, pneumonia, MDR, carbapenem resistance, inappropriate empirical antibiotics, and APACHE II score were each associated with 30-day mortality (*P*<0.05). Multivariate analysis included the following variables: *A. baumannii* genospecies, APACHE II score, age, pneumonia, vascular catheter infection, inappropriate empirical antibiotics, MDR and carbapenem resistance. The independent risk factors for 30-day mortality identified in the multivariate logistic regression analysis included *A. baumannii* genospecies (OR, 3.60; 95% CI, 1.56–8.33), pneumonia, age, and APACHE II score (Table 2).

**Table 2:** Risk factors for 30-day mortality in 180 patients with *Acinetobacter* bacteremia.

Variables	Univariate analysis			Multivariate analysis	
	No.(%) of patients		*P* value	Odds ratio (95% CI)	*P* value
	Survivor(n = 131)	Non-survivor (n = 49)			
Age^a^	55±18	69±12	<0.001	1.07 (1.03–1.10)	<0.001
Vascular catheter infection	44 (34)	10 (20)	0.086		
Pneumonia	23 (18)	22 (45)	<0.001	2.72 (1.16–6.37)	0.021
APACHE II score^a^	16±7	22±7	<0.001	1.12 (1.06–1.19)	<0.001
MDR	62 (47)	36 (74)	0.002		
Carbapenem resistance	38 (29)	27 (55)	0.001		
Inappropriate empirical antibiotics	65 (50)	37 (76)	0.002		
*A. baumannii* genospecies	55 (42)	35 (71)	<0.001	3.60 (1.56–8.33)	0.003

APACHE, acute physiology and chronic health evaluation; MDR, multidrug resistance.

a Continuous variables were expressed as means ± SDs and were compared by the Student’s *t* test.

**Figure 1:**
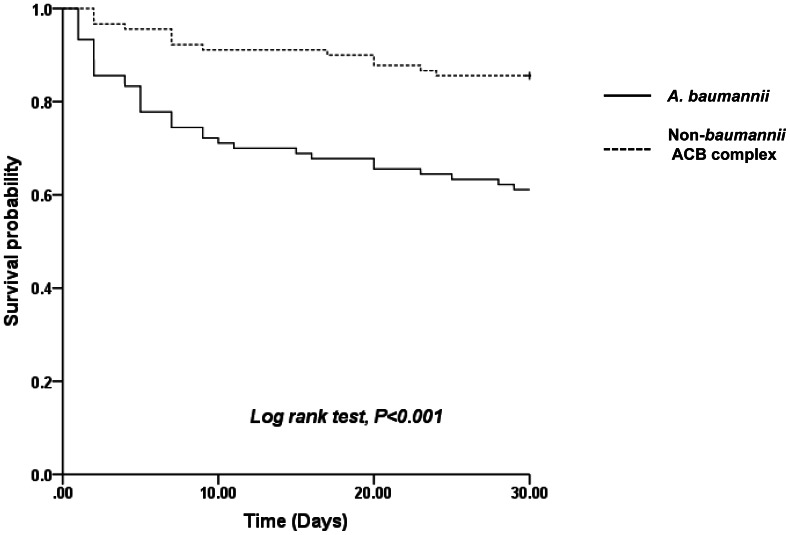
Kaplan-Meier curves of survival in 90 patients with *A. baumannii* bacteremia and 90 patients with non-*baumannii* ACB complex bacteremia.

The Kaplan-Meier survival curves for *Acinetobacter* bacteremia caused by *A. baumannii* and non-*baumannii* ACB complex strains are shown in Figure 1. The 30-day survival rate was 84% (72/90) in patients with non-*baumannii* ACB complex bacteremia compared to 61% (55/90) in those with *A. baumannii* bacteremia (log-rank test; *P*<0.001). No significant difference in survival was identified between patients with *A. nosocomialis* bacteremia (83%; 50/60), *Acinetobacter* species “close to 13 TU” bacteremia (88%; 15/17), and *A. pittii* bacteremia (91%; 10/11).

## Discussion

In this study, we found that genospecies *A. baumannii* was independently associated with poor outcome in patients with ACB complex bacteremia.

Non-*baumannii* ACB complex are increasingly reported as a cause of bacteremia because of the introduction of genospecies identification. Recent studies showed that *A. nosocomialis* and *A. pittii* comprised 27% and 9% of ACB complex bacteremia in Taiwan [13] and 21% and 8% in the USA [14], respectively, which is less prevalent than *A. baumannii*. However, *A. nosocomialis* and *A. pittii* were sevenfold more prevalent causes of bacteremia than *A. baumannii* in Norway [19]. In our study, the proportion of non-*baumannii* species was 50%, and *Acinetobacter* genomic species “close to 13 TU”, newly defined as a member of the ACB complex [9], comprised a higher proportion, in contrast to previous reports. This finding suggests that the distribution of prevalent genospecies differs geographically.

Previous studies reported the differences in clinical features between *A. baumannii* bacteremia and non-*baumannii* ACB complex bacteremia [12]–[14]. ICU onset, mechanical ventilation, higher Pitt bacteremia scores, and chronic obstructive pulmonary disease were factors associated with *A. baumannii* bacteremia, compared to non-*baumannii* ACB complex bacteremia in those studies. MDR, carbapenem resistance, and pneumonia were more commonly found in *A. baumannii* bacteremia, while primary bacteremia itself was more commonly caused by non-*baumannii* ACB complex bacteria. Similar results were found in our study. MDR was more commonly found in *A. baumannii* than non-*baumannii* ACB complex blood isolates, in agreement with our previous studies [15]. Additionally, we found chronic liver disease, which is prevalent in Korea, as another risk factor for *A. baumannii* bacteremia.

The relationship between genospecies and outcome of *Acinetobacter* bacteremia was evaluated in some studies; however, the results are inconsistent. In previous studies, which did not identify ACB complex bacteria to the species level with genotyping assays and did not include genotype as a confounding factor in analysis, carbapenem resistance was revealed as an independent risk factor for mortality in ACB complex bacteremia [20]. However, Chuang *et al*. reported that *A. baumannii* species, and not carbapenem resistance, was an independent predictor of mortality in ICU patients with *Acinetobacter* bacteremia in Taiwan [12]. However, another study performed at the same hospital during a similar period reported that bacteremia due to MDR strains, but not *A.* *baumannii per se*, appeared to be associated with a poor outcome [13].

In the current study, we also found that *A. baumannii* species, rather than antibiotic resistance, mainly affected mortality, in accordance with a previous [12] and a more recent study performed in the USA [14]. Recently, Peleg *et al*. suggested that *in vitro* and *in vivo* virulence characteristics differed among individual strains of the ACB complex [21], which provides further evidence of the impact of genospecies on the outcome of *Acinetobacter* bacteremia. Our data also suggest that genotype might be a more significant predictive factor for mortality than carbapenem resistance and that genospecies should be included as a confounding factor in any analysis of risk factors for mortality in patients with *Acinetobacter* infections, because genospecies is significantly associated with MDR and carbapenem resistance [22], [23] and can affect the outcome. Moreover, our data provide more evidence of the importance of correct genospecies identification, which can predict the outcome of *Acinetobacter* bacteremia.

Our study had several limitations. First, since it was retrospective in design, the factors influencing the physicians’ choice of antibiotics were not determined, and they may have influenced our results as unmeasured confounding factors in the analysis. Second, the current study was performed in a single center; for this reason, factors such as the rate of referrals or surgery for complex disorders could have impacted the results.

In conclusion, our data showed that *A. baumannii* genospecies is an independent predictor of mortality in patients with ACB complex bacteremia, emphasizing the importance of genotyping for correct species identification and prognosis prediction.
